# Cortical inhibition in neurofibromatosis type 1 is modulated by lovastatin, as demonstrated by a randomized, triple-blind, placebo-controlled clinical trial

**DOI:** 10.1038/s41598-022-17873-x

**Published:** 2022-08-15

**Authors:** Inês Bernardino, Ana Dionísio, Miguel Castelo-Branco

**Affiliations:** 1 Faculty of Medicine, Univ Coimbra, Pólo das Ciências da Saúde, Azinhaga de Santa Comba, 3000-548 Coimbra, Portugal; 2 Coimbra Institute for Biomedical Imaging and Translational Research (CIBIT), Univ Coimbra, Pólo das Ciências da Saúde, Azinhaga de Santa Comba, 3000-548 Coimbra, Portugal; 3 Institute of Nuclear Sciences Applied to Health (ICNAS), Univ Coimbra, Pólo das Ciências da Saúde, Azinhaga de Santa Comba, 3000-548 Coimbra, Portugal

**Keywords:** Developmental disorders, Inhibition-excitation balance

## Abstract

Neurofibromatosis type 1 (NF1) is associated with GABAergic dysfunction which has been suggested as the underlying cause of cognitive impairments. Previous intervention trials investigated the statins’ effects using cognitive outcome measures. However, available outcome measures have led to inconclusive results and there is a need to identify other options. Here, we aimed at investigating alternative outcome measures in a feasibility trial targeting cortical inhibition mechanisms known to be altered in NF1. We explored the neurochemical and physiological changes elicited by lovastatin, with magnetic resonance spectroscopy and transcranial magnetic stimulation (TMS). Fifteen NF1 adults participated in this randomized, triple-blind, placebo-controlled crossover trial (Clinicaltrials.gov NCT03826940) composed of one baseline and two reassessment visits after lovastatin/placebo intake (60 mg/day, 3-days). Motor cortex GABA+ and Glx concentrations were measured using HERMES and PRESS sequences, respectively. Cortical inhibition was investigated by paired-pulse, input–output curve, and cortical silent period (CSP) TMS protocols. CSP ratios were significantly increased by lovastatin (relative: *p* = 0.027; absolute: *p* = 0.034) but not by placebo. CSP durations showed a negative correlation with the LICI 50 ms amplitude ratio. Lovastatin was able to modulate cortical inhibition in NF1, as assessed by TMS CSP ratios. The link between this modulation of cortical inhibition and clinical improvements should be addressed by future large-scale studies.

## Introduction

Neurofibromatosis type 1 (NF1) consists of a multisystem neurodevelopmental disorder characterized by diverse clinical manifestations including pigmentary lesions, skeletal abnormalities, neurofibromas and learning difficulties^1,2^. Although the cognitive phenotype in NF1 is well characterized, the nature of its pathophysiology remains controversial. A long-standing hypothesis is based on the alterations in the gamma-aminobutyric acid (GABA) neurotransmission. Pioneering work in the *Nf1*^+/−^ mice model showed that Ras modulation by neurofibromin, a protein encoded by the *Nf1* gene, has a pivotal role in explaining learning and memory deficits^3^. Increased Ras activity found in the *Nf1*^+/−^ mice was associated with increased GABA-mediated inhibition, impairments in long-term potentiation (LTP) and learning deficits^3,4^. The translation of these findings to the human model has been performed by studies using in vivo magnetic resonance spectroscopy (MRS). In previous works from our group, reduced GABA concentration was consistently found both in children and adults with NF1, across different brain regions related with functional alterations described in these patients, namely in occipital and medial frontal cortices suggesting a compensatory mechanism^5–7^. Interestingly, medial frontal GABA levels were correlated with intellectual functioning and inhibitory control, supporting a role of GABA neurotransmission in cognitive functioning^8,9^. GABAergic system dysfunction in NF1 patients was also found to extend to the postsynaptic compartment, as assessed by GABA_A_ receptor binding potential, in the parieto-occipital cortex, midbrain, and thalamus corroborating the presence of both pre- and post-synaptic alterations^7^.

The increasing knowledge on the putative mechanisms underlying NF1 pathophysiology has motivated the investigation of potential therapeutic strategies targeting Ras activity, both in animal and human models. In preclinical studies, pharmacological intervention with lovastatin, a specific inhibitor of three-hydroxy-3-methylglutaryl coenzyme A (HMG-CoA) reductase, decreased the enhanced brain Ras activity, rescued LTP impairments, and improved spatial learning and attention deficits^10^. These promising results have not yet been translated by statin trials in humans, partly because of the difficulties in identifying sensitive and comparable outcome measures. Concerning cognitive improvements, preliminary evidence of potential benefits of lovastatin in memory, attention and emotional measures^11–13^ contrasts with no significant effect of treatment with statins reported in a substantial part of the studies^14–17^. Importantly, most part of research has been focusing on cognitive and behavioral outcome measures that frequently lack sensitivity and test–retest reliability^18^. Poor correlation between cognitive and behavioral measures was observed^19^ as well as low convergent validity between neuropsychological tests and functional indices, which are typically selected as outcome measures^20^. This stresses the clear need to explore alternative outcome measures to evaluate novel therapeutic approaches targeting identified mechanisms of disease^18^.

Here, we adopted a multimodal approach combining MRS and transcranial magnetic stimulation (TMS), both non-invasive in vivo techniques that have been used to study cortical inhibition and GABAergic activity^21^. There is evidence that each technique targets specific aspects of GABAergic neurotransmission^22,23^, suggesting a complementary role. TMS is associated with synaptic activity, while GABA levels obtained from MRS reflect tonic instead of phasic inhibitory processes^24^. We hypothesize that measures that address physiological mechanisms known to be altered in this clinical model might be more sensitive to investigate whether inhibitory deficits at the synaptic level may be altered by lovastatin^13^, since they precede behavioral changes, producing earlier measurable outcomes^6,25^.

Thus, we aimed to investigate acute physiological effects following lovastatin intake (neurophysiological and neurochemical changes), in patients with NF1 by designing an exploratory interventional study based on the critical link between mechanisms of disease, and well-defined therapeutic targets.

We conducted a clinical trial, comprising three visits, wherein we performed TMS and MRS at baseline, and then repeated the protocol in the subsequent sessions, each following a 3-day course of lovastatin/placebo administration.

## Results

### Transcranial magnetic stimulation (TMS)

**Figure 1:**
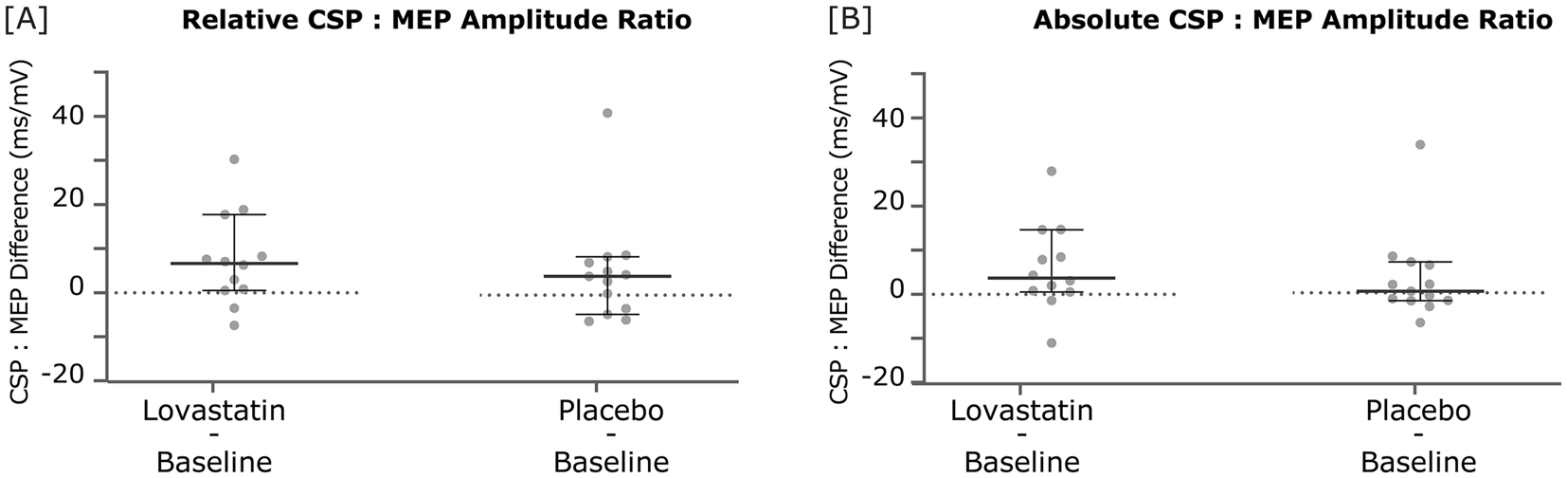
Differences in relative (**A**) and absolute (**B**) cortical silent period ratios, comparing measures taken after lovastatin and placebo intake with the baseline assessment. Dots represent, for each participant, the difference between lovastatin and baseline or placebo and baseline CSP:MEP ratios. Lines represent median and 95% CI. *CSP* cortical silent period, *MEP* motor-evoked potential, *ms* millisecond, *mV* millivolt, *CI* confidence interval.

Cortical Silent Period revealed a significant increase in the normalized relative and absolute CSP duration:MEP amplitude ratios following lovastatin (relative: *Z* = − 2.197, *p* = 0.027, n = 12; absolute: *Z* = − 2.118, *p* = 0.034, n = 12) and not placebo (relative: *Z* = − 1.433, *p* = 0.168, n = 13; absolute: *Z* = − 0.943, *p* = 0.376, n = 13) administration (Fig. 1, and Supplementary Fig. S1). CSP measurements were independent from the force exerted during this stimulation protocol (20% of maximum muscle contraction), as attested by the lack of correlation between them (*p* ≥ 0.655).

**Figure 2:**
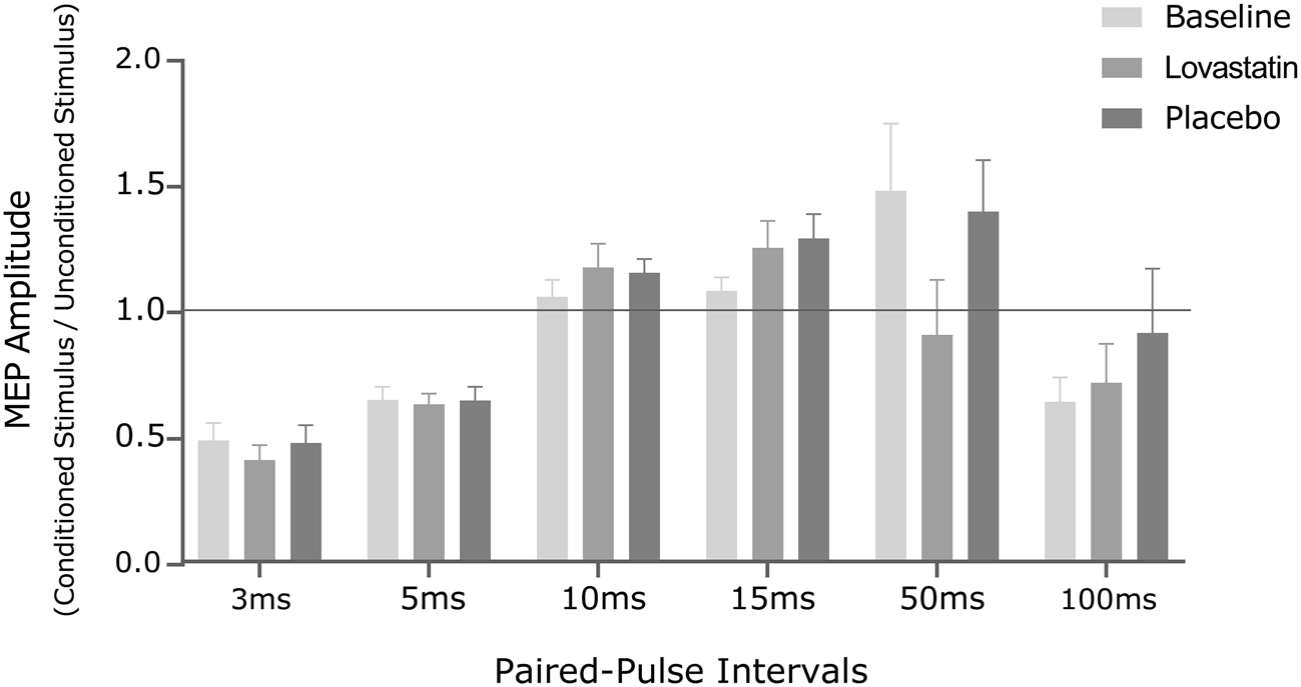
MEP peak-to-peak amplitude ratios for SICI3ms, SICI5ms, ICF10ms, ICF15ms, LICI50ms and LICI100ms, at baseline and after lovastatin and placebo administration. The horizontal line represents a null effect, wherein conditioned stimulus amplitude equals the amplitude from baseline pulses. Inhibition occurs for bars below the horizontal line, whereas excitation stands above the line.* MEP* motor-evoked potential, *ms* millisecond.

Concerning the paired-pulse paradigm, we detected a trend for inferior amplitude ratios in LICI 50 ms after lovastatin that was not observed after placebo (lovastatin: *Z* = − 2.023, *p* = 0.063, n = 5; placebo: *Z* = − 0.365, *p* = 0.875, n = 4; Fig. 2). For the remaining intervals significant differences were not observed (*p* ≥ 0.104).

**Figure 3:**
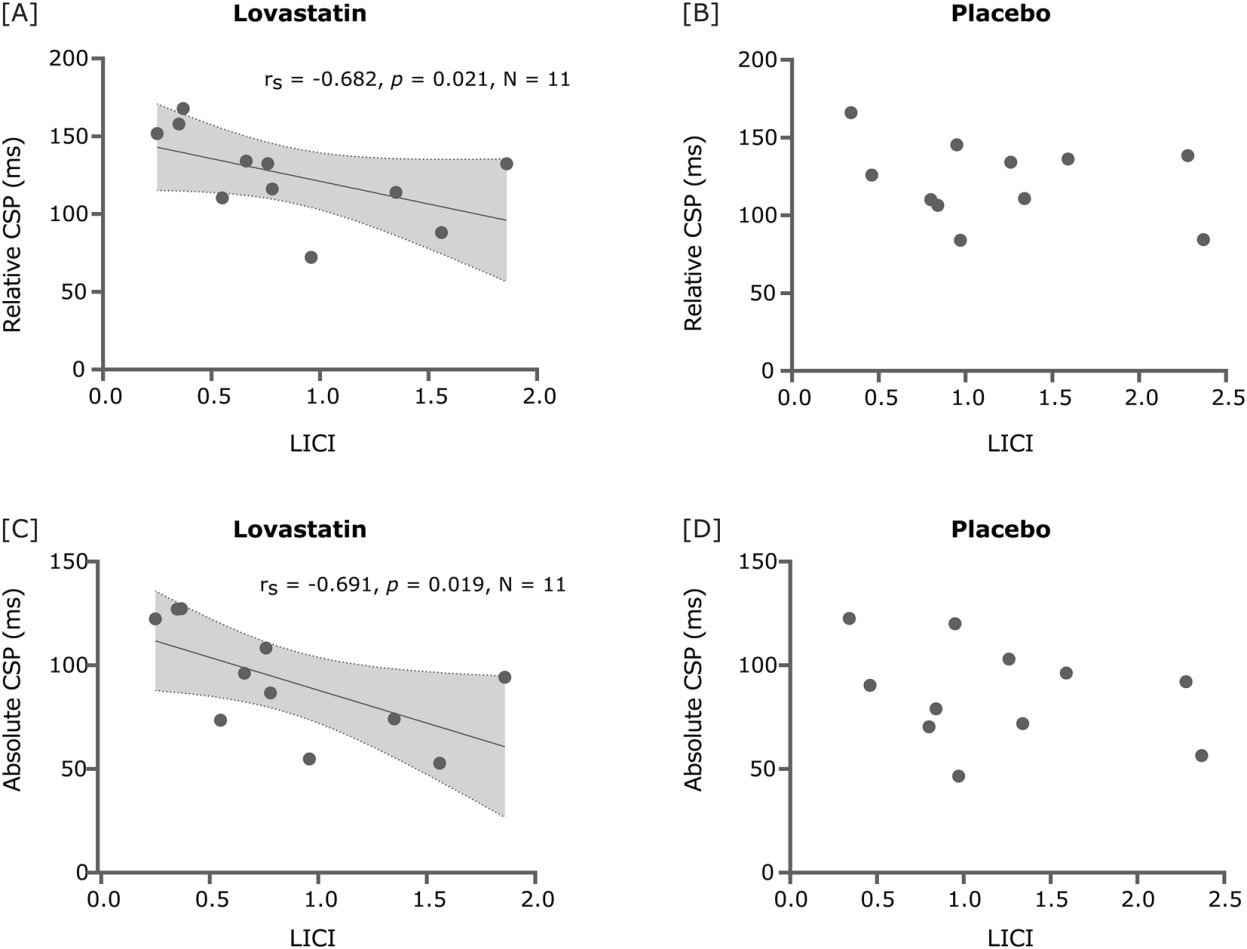
Spearman correlations between relative and absolute silent periods and MEP amplitude ratio in LICI mean intervals, both following lovastatin (**A**, **C**) and placebo (**B**, **D**). Shaded area represents the 95% CI for the best-fit line. *CSP* cortical silent period, *ms* millisecond, *CI* confidence interval, *LICI* long-interval intracortical inhibition.

Additionally, we checked for correlations between GABA_B_-related measures (CSP and LICI) and observed significant negative moderate correlations between MEP amplitude ratio in LICI (mean of 50 ms and 100 ms intervals) and both relative (lovastatin: r_s_ = − 0.682, *p* = 0.021, n = 11; placebo: r_s_ = − 0.182, *p* = 0.593, n = 11) and absolute (lovastatin: r_s_ = − 0.691, *p* = 0.019, n = 11; placebo: r_s_ = − 0.264, *p* = 0.433, n = 11) CSP durations for the lovastatin condition (Fig. 3).

The input–output protocol did not reveal significant differences in the curve slope (*p* ≥ 0.413, n = 11), maximum MEP amplitude (*p* ≥ 0.577, n = 11) or S50 (*p* ≥ 0.497, n = 13).

### Proton magnetic resonance spectroscopy (^1^H MRS)

The voxel overlap between the assessment points was confirmed by the consistent tissue composition (GM, WM and CSF), in comparison with the baseline (*p* ≥ 0.165; Table 1).

**Table 1:** MRS voxel tissue proportions.

	Baseline assessment		Lovastatin		Placebo	
	Mean (S.E.)	Range	Mean (S.E.)	Range	Mean (S.E.)	Range
GM	0.27 (0.005)	0.25–0.31	0.28 (0.008)	0.24–0.31	0.27 (0.006)	0.22–0.31
WM	0.63 (0.013)	0.55–0.71	0.63 (0.017)	0.52–0.72	0.64 (0.011)	0.58–0.71
CSF	0.10 (0.013)	0.04–0.17	0.09 (0.013)	0.04–0.18	0.08 (0.007)	0.06–0.15

*S.E.* standard error, *GM* gray matter, *WM* white matter, *CSF* cerebrospinal fluid.

MRS spectra selected for analysis revealed good quality as verified by Fit Error and Cramér-Rao parameters for HERMES and PRESS sequences, respectively. Moreover, FWHM and SNR showed no significant differences between sessions (*p* ≥ 0.119; Table 2).

**Table 2:** MRS data quality parameters.

	Baseline assessment		Lovastatin		Placebo	
	Mean (S.E.)	Range	Mean (S.E.)	Range	Mean (S.E.)	Range
**HERMES**						
Fit Error (%)	6.30 (0.45)	3.31–8.15	6.77 (0.42)	4.13–8.91	7.36 (0.55)	4.86–9.68
FWHM (Hz)	10.43 (0.67)	8.97–15.50	10.00 (0.46)	7.69–12.57	9.52 (0.46)	7.87–13.24
SNR	10.05 (0.75)	7.15–15.09	8.52 (0.61)	5.36–10.76	8.05 (0.49)	6.04–10.06
**PRESS**						
CRLB (%)	7.18 (0.263)	6–9	7.62 (0.21)	7–9	7.42 (0.23)	6–9
FWHM (Hz)	6.39 (0.53)	4.22–9.71	5.94 (0.36)	4.22–8.56	5.88 (0.50)	4.22–10.99
SNR	40 (2.59)	30–58	40.46 (1.54)	34–50	35.42 (1.68)	28–49

*S.E.* standard error, *FWHM* full width at half maximum, *SNR* signal-to-noise ratio, *CRLB* Cramér-Rao Lower Bound.

We observed a trend for a reduction in absolute Glx concentration after lovastatin intake (lovastatin: *t* = 2.017, *p* = 0.074, n = 10; placebo: *t* = 0.285, *p* = 0.782, n = 10), but not in Glx tissue-corrected levels (lovastatin: *t* = 1.125, *p* = 0.293, n = 9; placebo: *t* = 1.717, *p* = 0.130, n = 8). Baseline GABA+ concentrations were not significantly altered following lovastatin (*t* = − 0.275, *p* = 0.791, n = 9) or placebo administration (*t* = − 0.838, *p* = 0.430, n = 8).

## Discussion

The current work provides the first randomized, triple-blind, placebo-controlled trial combining MRS and TMS techniques, in the same NF1 group, to investigate whether outcome measures based on cortical inhibitory dysfunction may be modulated by lovastatin. Previous statin trials have failed at definitively establishing the effect of treatment in this population^11–17^. This may be due to the previous choice of available outcome measures. Here, we proposed a feasibility study testing outcomes at the neural system level by investigating the effects of treatment from the neurochemical and physiological points-of-view.

Considering physiological outcomes, we did observe a significant increase in the absolute and relative CSP:MEP amplitude ratios, consistent with the hypothesis of increased intrahemispheric inhibition following lovastatin. The corrected measure allowed us to address CSP intrinsic variability, when studying the corticospinal pathway’s net excitability^26^. Importantly, the muscle contraction applied during this paradigm did not influence CSP results as attested by the absence of significant correlations between these measures^27^. The origin of the CSP is still under debate. Although some authors claim that spinal processes may also play an important part, the literature points out a major role of cortical mechanisms through the recruitment of inhibitory interneurons in the cortex^26,28^. The CSP paradigm has been demonstrated to be valuable in studying inhibition of the motor cortex, particularly GABA_B_-mediated alterations^26–28^.

Regarding the paired-pulse protocol, we observed a trend towards a decrease in peak-to-peak amplitude of motor-evoked potentials ratio for an ISI of 50 ms, suggesting superior long-interval intracortical inhibition after lovastatin intake. Interestingly, although at baseline NF1 patients did not show an inhibition for LICI 50 ms interval, unlike healthy individuals, possibly resulting from counteracting facilitatory mechanisms^29^, after lovastatin the expected inhibition was observed. This effect at a neurophysiological transition zone between excitation and inhibition might also reinforce a tendency for lovastatin to modulate the excitatory-inhibitory imbalance. This was corroborated by the only study we found including TMS outcome measures to address statin intervention in NF1^13^, wherein modulation of inhibition by lovastatin intake was also detected in another paired-pulse inhibitory protocol, particularly in SICI intervals. In our study, however, SICI and ICF were not altered, suggesting an effect of lovastatin in paired-pulse GABA_B_-mediated measures, namely LICI.

In fact, our main findings included LICI and CSP measures, both being associated to GABA_B_ receptor, with CSP measuring the duration and LICI assessing the magnitude of cortical inhibition^30^. After lovastatin, we found a significant moderate negative correlation between the duration of absolute and relative silent periods and the MEP ratio in LICI (mean of 50 and 100 ms ISIs), that was not observed in placebo. A longer silent period correlated with an inferior peak-to-peak amplitude of MEPs in the LICI paradigm, both indicating an increase of inhibition. Even though a correlation was present between CSP and LICI after lovastatin, this only explains a part of the variance, so we may suggest that there are other components unrelated to GABA_B_ that could explain the variance in these measures, rendering a null correlation for the placebo condition.

Regarding our MRS findings, a trend towards a decrease in absolute Glx in motor cortex was observed after lovastatin intake, whereas GABA+ concentrations were not altered. The Placebo condition revealed no effect. The Glx findings are in line with those reported in a trial, in NF1 young children, showing reduced gray nuclei Glx after treatment with simvastatin^15^ and support preliminary evidence that statins may introduce changes in the excitatory-inhibitory imbalance described in NF1^5,7^.

Taken together, our findings suggest that some measures of TMS, namely CSP and, into a lesser extent paired-pulse stimulation, demonstrated higher sensitivity than MRS to detect changes in the GABAergic system induced by lovastatin, since each one measures specific aspects of GABAergic neurotransmission^22,23^. It is worth mentioning that a reliable biomarker should be able to detect statistically significant differences while minimizing the presence of dropouts. Taking this into account, we do not recommend future studies to rely on LICI measures since the lower signal-to-noise ratio highly impacts the amount of good-quality data for analysis.

An additional purpose of this study was to investigate potentially more sensitive outcome measures, trying to overcome the lack of prior efficient detection of lovastatin effects in NF1 excitation-inhibition imbalance as well as in behavioral measures. Although the effectiveness of lovastatin in rescuing NF1 symptoms can only be assessed in larger trials exploring the link between the modulation of physiological cortical inhibition measures and clinical symptoms, our study sheds some light on the selection of more suitable outcome measures for future Phase 2b/III clinical trials, highlighting the potential of cortical silent period TMS measures.

It is important to take some limitations into consideration when interpreting our results. Due to the methodological demands associated with this challenging feasibility study design, the sample size was relatively small and the amount of data eligible for analyses was impacted by the rigorous quality criteria employed. This takes additional caution given MEPs’ variability^25^. Additionally, we did not include a healthy control group for baseline measurements since we adopted a cross-over experimental within-subject design. Our approach targeted physiological and neurochemical measures and, therefore, we cannot infer about the alterations in cognitive and behavior domains reported in these patients. In this work, we studied cortical inhibition in the motor cortex, which has been barely explored in NF1^27^ and, therefore, we cannot generalize our results about the effects of lovastatin to other areas of the brain. In fact, since GABA levels were found to be altered in occipital and medial frontal cortices^5,6^, future studies should also target these regions to investigate whether neurochemical alterations can be rescued by lovastatin. The lovastatin dosage was established based on safety criteria from common use in other pathologies such as hypercholesterolemia, and we also selected a shorter duration treatment, when comparing with other studies^14–17^, targeting earlier neurochemical and physiological changes in the GABAergic system, while enhancing patients’ compliance.

This study was performed with the aim of investigating physiological acute effects elicited by lovastatin intervention, using synaptic inhibition outcome measures. An objective physiological approach may provide evidence of early changes that can precede alterations in high-level cognitive and behavioral outcome measures. We observed alterations in the excitatory-inhibitory push–pull mechanism in NF1 patients, induced by lovastatin as measured by the CSP stimulation protocol. These altered mechanisms and the link with the clinical symptoms observed in NF1 patients should be further explored in future large-scale studies in which TMS-based cortical silent period measures seem to represent an important asset to evaluate the lovastatin-induced effects in the treatment of neurological conditions, such as NF1.

## Methods

### Study design

A randomized, triple-blind, placebo-controlled Phase 2a Clinical Trial, first registered on 01/02/2019, at clinicaltrials.gov, with the registration number #NCT03826940 (ASD/NF1inhib), was conducted from 19/02/2019 to 31/08/2020, with the main goal to investigate the physiological and neurochemical effects of lovastatin in a group of adults with NF1, while addressing the sensitivity of TMS and MRS as outcome measures. All subjects were allocated to both real and placebo intervention following a crossover design. Study procedures were reviewed and approved by the local Ethics Committees of the Faculty of Medicine of the University of Coimbra and performed in accordance with the Declaration of Helsinki. All participants provided written informed consent prior to their inclusion.

### Participants

A total of 17 patients with NF1 were sampled from a database as in previous studies^5,6^ and with the collaboration of the national NF1 patients association (APNF). Two participants were not able to initiate the experimental procedures. As a result, a total of 15 NF1 patients, aged between 26 and 55 years (mean ± SE = 39.60 ± 2.08 years), were enrolled in this study carried out at the CIBIT/ICNAS facilities (University of Coimbra, Portugal). All patients met the National Institute of Health (NIH, Consensus Development Conference 1988^31^) diagnostic criteria for NF1. Exclusion criteria were as follows: other neurological or psychiatric disorders, abnormal liver function, history of traumatic brain injury, epilepsy, substance abuse, contraindications to MR scanning or TMS, severe learning disabilities (WAIS-III, IQ < 70), previous or current statin use and usage of medication with potential interaction with lovastatin. All participants received an abbreviated form of the Portuguese adapted version of the Wechsler Adult Intelligence Scale, 3rd edition^32^. Handedness was assessed using the Edinburgh Inventory^33^. The demographic characteristics are summarized in Table 3.

**Table 3:** Demographic characteristics.

	NF1 participants (*n* = 15)	
	Mean (S.E.)	Range
Chronological-age (years)	39.60 (2.08)	26–55
Level of education (years)	12.73 (0.90)	4–17
Full-scale IQ	102.80 (3.49)	76–128
Sex (male : female)	6:9	
Handedness (right : left)	14:1	

*NF1* neurofibromatosis type 1, *S.E.* standard error, *IQ* intelligence quotient.

### Medication protocol and randomization

The order of lovastatin or placebo intake was randomized for each participant, following a computer-generated permutation-block 1:1 randomization list. Sequentially numbered containers were used. Randomization was managed by the Clinical Research Unit of the University of Coimbra, which performed patient assignment and drug distribution. Patients and all study investigators were blinded to treatment allocation. The authors were blinded until all data analyses were completed.

Participants were treated with 60 mg per day (3 pills of 20 mg) of either lovastatin or placebo (both with the same color, size and shape) for 3 consecutive days, at night. Adverse events and study compliance were assessed when patients returned for reassessment. Monitorization during the intake period was conducted by phone contact. Participants were not able to distinguish between lovastatin and placebo conditions and reported no lovastatin-related symptoms.

### Procedures and outcome measures

**Figure 4:**
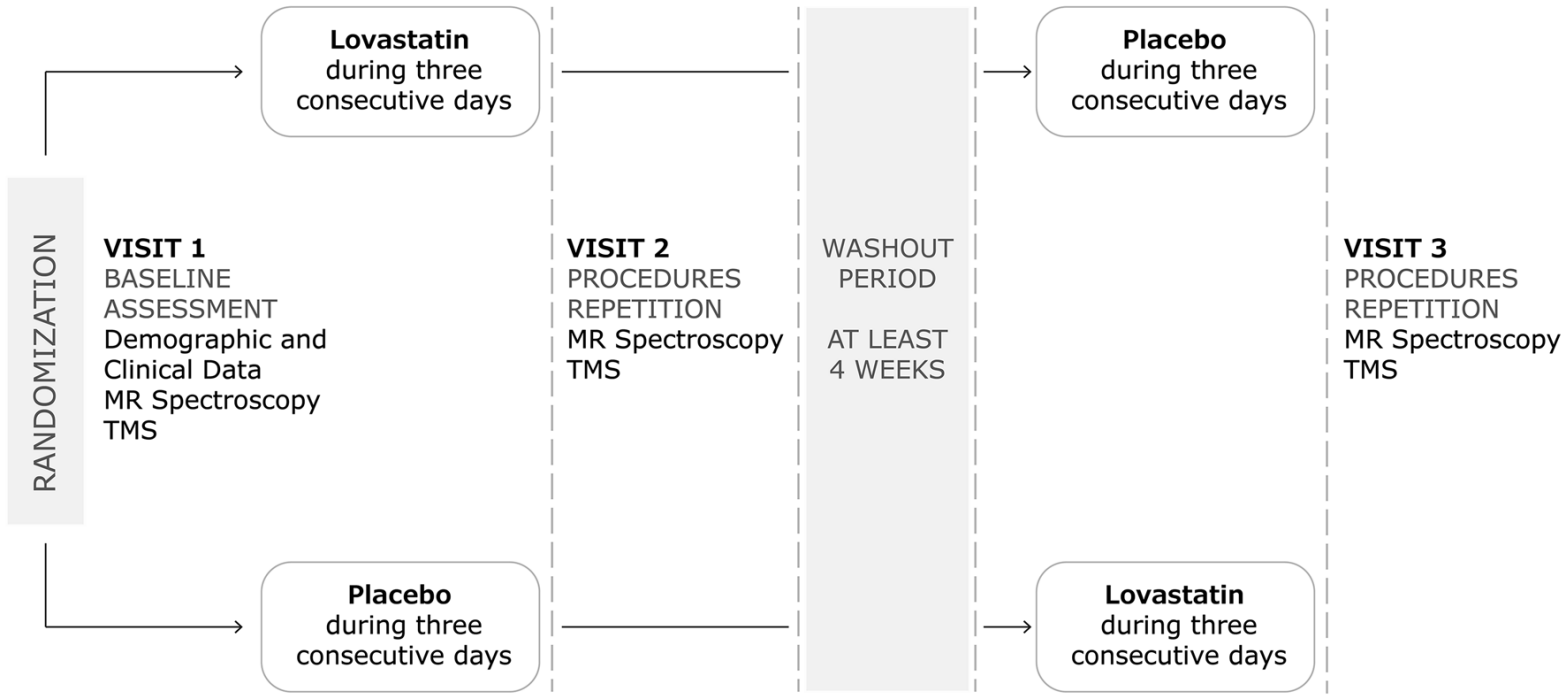
Study design, including the procedures performed in each visit. *MR* magnetic resonance, *TMS* transcranial magnetic stimulation.

The study protocol (Fig. 4) encompassed 3 visits. The first comprised the baseline assessment. Participants underwent screening procedures, intellectual functioning assessment and MRS followed by TMS. After this session, participants took lovastatin or placebo during three consecutive days and returned, in the immediate next day, for a second visit in which all assessments, but intellectual evaluation, were repeated. After a washout period of 4 weeks minimum (range: 33–110 days, mean: 53.27 ± 24.15 days) participants took three days of placebo or lovastatin (cross-over design) and returned, again in the 4th day, to repeat all the assessments (third visit). All procedures were performed at the same time of the day across sessions, within- and between-subjects.

Outcome measures were assessed at the 3 visits. These included the peak-to-peak amplitude of motor-evoked potentials as well as durations of cortical silent periods, evaluated through TMS, and GABA and Glx concentrations, obtained from MRS.

### Transcranial magnetic stimulation (TMS)

Participants were comfortably seated in an armchair, equipped with a head support to achieve better immobilization. We used a MCF-B65 figure-of-eight coil (MagVenture, Denmark) connected to a MagPro X100 magnetic stimulator (MagVenture, Denmark). The coil was positioned over the dominant primary motor cortex, at 45° to the sagittal plane, with a posterior-to-anterior direction, in order to achieve optimal resting motor thresholds (rMT)^26^. After finding the hotspot, we delineated the positioning of the coil in a swimming cap to ensure the same localization and orientation in all protocols and across sessions.

A BIOPAC MP-150 system, equipped with an EMG 100C amplifier (Biopac Systems, CA, USA), with a gain of 1000, was used to record motor-evoked potentials (MEPs). Prior to the recording, skin was prepared with Nuprep and alcohol, and Ag/AgCl electrodes (Biopac Systems, CA, USA) placed in a belly-tendon montage in first dorsal interosseous (FDI) muscle, were filled with conductive paste. The electromyography (EMG) signal was then acquired and processed in the Acqknowledge 4.2 software (Biopac Systems, CA, USA), with a 2.5 kHz sampling rate. A quality inspection was performed to ensure only data with at least 5 valid and measurable motor-evoked potentials were included in the analyses, for each protocol which rendered a minimum of 11 participants in each analysis except for LICI (minimum of 4) due to its low signal to noise ratio.

### Paired-pulse (pp-TMS)

For the paired-pulse protocols, we adjusted the intensity until we had achieved the lowest stimulus intensity required to evoke MEPs with minimum peak-to-peak amplitude of 1 mV (SI1mV), in at least 5 out of 10 consecutive trials.

Short-interval intracortical inhibition (SICI) and intracortical facilitation (ICF) were assessed by the application of a subthreshold (80% SI1mV) conditioning stimulus followed by a suprathreshold (120% SI1mV) test stimulus, for inter-stimulus intervals (ISIs) of 3 ms and 5 ms for SICI and of 10 and 15 ms for ICF, each ISI comprising 15 trials. In each protocol, 10 baseline single-pulses at 120% SI1mV were applied. Moreover, for long-interval intracortical inhibition (LICI) we applied 30 paired-pulses with ISIs of 50 and 100 ms, with both conditioning and test stimuli set at 100% SI1mV, and 10 single-pulses with the same intensity. For all paradigms, pulses were administered in a randomized order and with random intervals between pairs of pulses, ranging from 7 to 11 s.

The detection of motor-evoked potentials and the measurement of its amplitudes were performed with an in-house script and confirmed, trial-by-trial, through visual inspection by two blinded authors. We determined paired-pulse:baseline MEP amplitude ratios, for each subject.

### Input–output or recruitment curve (I–O curve)

Regarding input–output curve, we recorded MEPs at six different intensities: 90% to 140% rMT with increments of 10%, as in De Beaumont et al.^34^. To determine rMT, we adjusted the intensity until we had reached the smallest intensity required to evoke MEPs with minimum peak-to-peak amplitude of 50 µV in at least 5 out of 10 consecutive trials. We delivered 10 pulses at each intensity in a randomized order and with random inter-stimulus intervals (from 6 to 8 s). After constructing the curve, we extracted the curve slope, maximum MEP peak-to-peak amplitude and S50 (the stimulus intensity that evoked a half-maximal MEP).

### Cortical silent period (CSP)

We applied a suprathreshold pulse during voluntary muscle contraction of the dominant hand, producing a motor-evoked potential followed by a period of EMG silence. Measures were obtained while participant maintained a 10-s contraction (5 s before and another 5 s after TMS pulse) of 20% of the maximal force, measured with a hand-held digital dynamometer, which enabled participants to control *online* the force they were exerting. Ten CSPs were recorded through EMG using an intensity of 130% of rMT. A 10-s resting period between contractions was included in order to prevent fatigue, which could potentially interfere with intracortical inhibition mediated by GABA_B_^35^. Both absolute and relative silent periods were marked manually by visual inspection and double-checked by two blinded authors, as in Säisänen et al.^36^. We normalized duration measures by determining CSP:MEP ratios, as suggested by Hupfeld et al.^26^, to mitigate interindividual differences. Breakthrough EMG activity was considered as being part of the cortical silent period, and therefore included in the period measures, as recommended by Hupfeld et al.^26^.

### Proton magnetic resonance spectroscopy (^1^H MRS)

Magnetic resonance experiments were carried out at CIBIT/ICNAS facilities (University of Coimbra), on a magnetic resonance imaging scanner (Siemens 3 T MAGNETOM Prisma Fit, Erlangen, Germany), equipped with a 20-channel birdcage head coil. A high-resolution T1-weighted 3D MPRAGE (Magnetization Prepared Rapid Acquisition Gradient Echo) sequence 1 × 1 × 1 mm^3^ was acquired to obtain anatomical data. The following parameters were used: repetition time (TR) = 2530 ms, echo time (TE) = 3.50 ms, inversion time (TI) = 1100 ms, flip angle (FA) = 7°, field of view (FOV) = 256 × 256 mm^2^, 176 slices. Then, participants performed a finger-tapping task as a functional localizer to determine the motor area for MRS voxel placement. The finger-tapping task consisted in a previously validated paradigm, as detailed in Silva et al.^37^, wherein the participant performed synchronous or asynchronous tapping, depending on the visual cue, at different frequencies.

**Figure 5:**
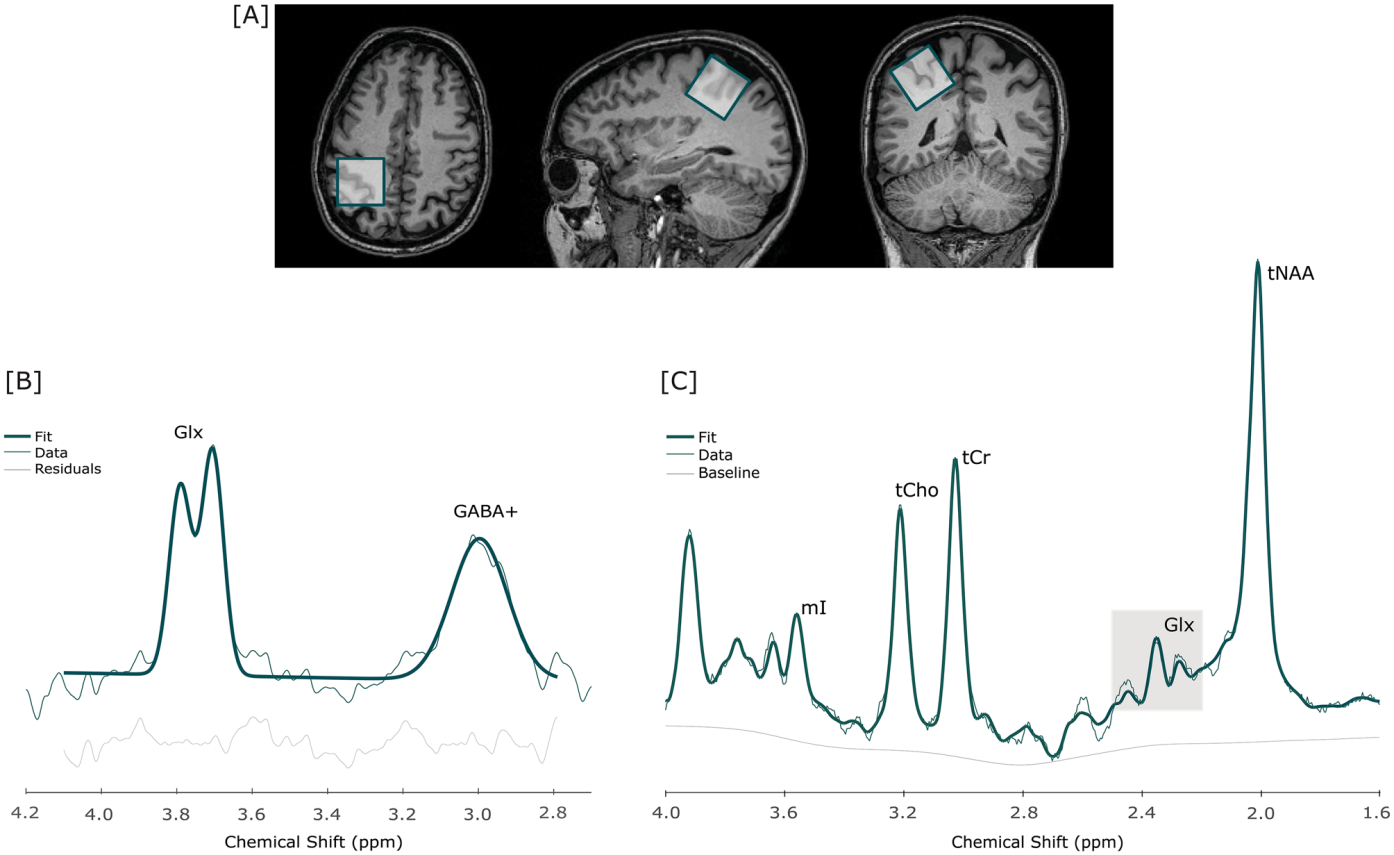
A schematic representation of the voxel placement (**A**). In (**B**), it is presented an example of Gannet output, from the HERMES sequence, used to estimate GABA+ concentration. Glx levels were quantified through the PRESS sequence acquisition, processed in LCModel, as represented by the example spectrum (**C**). *Glx* glutamate + glutamine, *GABA+* gamma-aminobutyric acid, *mI* myo-inositol, *tCho* total choline, *tCr* total creatine, *tNAA* total *N*-acetylaspartate, *ppm* parts per million.

We positioned a 3 × 3 × 3 cm^3^ voxel in the dominant motor cortex to estimate GABA and Glx levels in the selected volume (Fig. 5). The voxel positioning was replicated in all sessions, for each participant, by saving the anatomical location and orientation of the voxel from the first (baseline) visit and using it as a reference for the subsequent sessions.

To assess GABA content, we applied the HERMES (Hadamard Encoding and Reconstruction of MEGA-Edited Spectroscopy) sequence^38,39^, using: TR = 2000 ms, TE = 80 ms, FA = 90°, 320 averages and 2048 points. We applied two editing frequency-selective inversion pulses at 1.90 and 4.56 ppm and a “off resonance” pulse at 7.50 ppm. Additionally, we also acquired spectra with no water suppression (32 averages), within the same delineated volume-of-interest.

Glx concentration was estimated through the acquisition of the PRESS (Point RESolved Spectroscopy) sequence in the same voxel used for HERMES, with TR = 2000 ms, TE = 35 ms, FA = 90°, 46 averages and 1024 points. Moreover, we acquired spectra without water suppression (16 averages).

### GABA quantification

We used Gannet GABA Analysis Toolkit, version 3.1.5, in MATLAB (R2020b, MathWorks, USA) to quantify GABA in the motor cortex. We have applied an exponential line broadening at 3 Hz. Eddy current correction was applied on water and metabolite data and residual water signal was removed from the difference spectra by an HSVD filter. For frequency and phase correction, RobustSpecReg alignment was selected, as recommended in the toolkit. Two blinded authors evaluated the quality of the spectra by visual inspection. We established a cut-off of 10% for maximum fit error and analyzed full width at half maximum (FWHM) and signal-to-noise ratio (SNR). Poor quality spectra were not included in the analyses. Data from a minimum of 8 participants were analyzed. Due to the possibility of GABA levels having a contribution from macromolecules and homocarnosine^40^, here, we will designate this metabolite as GABA+. We performed coregistration and segmentation with Gannet and SPM12 toolbox (Wellcome Trust Centre for Neuroimaging, Institute of Neurology, UCL, London, UK) to assess the percentage of gray matter (GM), white matter (WM) and cerebrospinal fluid (CSF) in the voxel and used alpha tissue correction, a method by Harris et al.^41^, which allowed us to correct GABA+ concentration for voxel tissue composition.

### Glx quantification

Data from PRESS sequence were processed in LCModel v. 6.3-1 M^42^. We applied eddy-current correction and water scaling. We analyzed spectra with chemical shift from 1.6 to 4.0 ppm, minimizing lipid and macromolecules artifacts. The quality of data was evaluated by two blinded investigators who visually inspected all spectra and excluded those with poor quality or Crámer-Rao Lower Bounds (CRLB) superior to 10%. Additionally, we analyzed FWHM and SNR. Data from 10 participants were eligible for analyses. Glx concentration was corrected for tissue composition (GM, WM and CSF) through the method described in Naaijen et al.^43^.

### Statistical analyses

Every participant returned for follow-ups, completing all the procedures. No intake was missed by any participant, even though one patient took 40 mg out of 60 mg on the first day, and the full 60 mg dosage on the next 2 days. We, therefore, achieved 99.3% of compliance by counting returned pills. A conservative intention-to-treat analysis of all data was performed. Statistical analyses were carried out in SPSS Statistics v. 27 (IBM SPSS Statistics, IBM Corporation, Chicago, IL). We adopted a significance level of 0.05. Descriptive statistics were applied to characterize our sample regarding demographic data. Normality of the data was assessed with the Shapiro–Wilk test and extreme outliers (defined as data points lower than Q1-3 × IQR or greater than Q3 + 3 × IQR) were excluded. Since TMS data were not normally distributed, we performed the equivalent non-parametric Wilcoxon test and reported exact *p*-values. Moreover, we checked for significant correlations between CSP and LICI with Spearman’s rho. Differences in GABA+ and Glx concentrations between baseline and post-intake assessments were computed with paired-sample t-tests.

### Ethics approval and consent to participate

Study procedures were reviewed and approved by the local Ethics Committees of the Faculty of Medicine of the University of Coimbra and performed in accordance with the Declaration of Helsinki. All participants provided written informed consent prior to their inclusion.

## Supplementary Information

Supplementary Figure S1.

## Data Availability

The datasets generated during and analysed during the current study are available from the corresponding author on reasonable request.
